# From Diversity Ideologies to the Expression of Stereotypes: Insights Into the Cognitive Regulation of Prejudice Within the Cultural-Ecological Context of French Laïcité

**DOI:** 10.3389/fpsyg.2020.591523

**Published:** 2021-01-12

**Authors:** Lucie-Anna Lankester, Theodore Alexopoulos

**Affiliations:** ^1^Laboratoire de Psychologie Sociale: Contextes et Régulation (EA 4471), Institute of Psychology, Université de Paris, Paris, France; ^2^Centre de Recherches sur la Cognition et l’Apprentissage (UMR 7295), Centre National de la Recherche Scientifique, Université de Poitiers, Poitiers, France

**Keywords:** diversity ideologies, ethnic stereotypes, cultural determinants, suppression process, justification process, French Laïcité, cultural norms of diversity

## Abstract

This theoretical paper examines the context-sensitivity of the impact of cultural norms on prejudice regulation. Granting the importance of understanding intergroup dynamics in cultural-ecological contexts, we focus on the peculiarities of the French diversity approach. Indeed, the major cultural norm, the Laïcité (i.e., French secularism) is declined today in two main variants: The Historic Laïcité, a longstanding egalitarian norm coexisting with its amended form: The New Laïcité, an assimilationist norm. In fact, these co-encapsulated Laïcité variants constitute a fruitful ground to cast light on the processes underlying prejudice regulation. Indeed, it is documented that the assimilationist New Laïcité is linked to higher levels of prejudice as compared to the egalitarian Historic Laïcité. To this day, research mainly explored interindividual determinants of Laïcité endorsements and specified how these endorsements shape prejudice. Crucially, this “indirect-endorsement path” does not account for the more straightforward causal relationship between Laïcité and prejudice. Moreover, recent experimental evidence suggests that the normative salience of both Laïcité norms shape intergroup attitudes beyond personal endorsement. Therefore, in this contribution, we complement previous work by investigating the possible socio-cognitive processes driving this “direct-contextual path.” In doing so, we seek to bridge the gap of causality by investigating how the Laïcité norms can set the stage for specific regulatory strategies. Our reasoning derives from an application of the Justification-Suppression Model bolstered by classical work on mental control, modern racism and diversity ideology. From this, we sketch out the operative functioning of two distinct regulation processes: (a) one that prevents prejudicial attitudes but which can have unexpected consequences on stereotyping within the Historic Laïcité context (i.e., suppression) and (b) one that helps realize prejudice within the New Laïcité context (i.e., justification). From this analysis, we discuss the consequences for intergroup relations within and beyond the French context. In particular, we outline the importance of an adequate framing of egalitarian ideologies so that they achieve their goal to foster harmonious intergroup relations.

## Introduction

One of the major hurdles to the development of harmonious intergroup relations within societies is the persistence of cultural stereotypes (i.e., shared beliefs about the attributes of outgroup members) which is a fertile ground for the endurance of racism within societies (Allport, 1954; Tajfel, 1969; Devine, 1989; Hamilton and Sherman, 1994; Park and Judd, 2005; Crandall et al., 2011). In fact, the expression of cultural stereotypes is fueled and driven by ethno-religious prejudice^1^ (i.e., negative evaluations of individuals based on their group membership; Crandall and Eshleman, 2003). To address this phenomenon, across cultures, political authorities rely on diversity ideologies, namely, belief systems regarding the ways society should approach ethno-religious diversity (Rosenthal and Levy, 2010; Levin et al., 2012). Depending on the country, diversity ideologies shape *cultural norms of diversity*^2^, namely, the shared and perceived national ways to deal with diversity (Guimond et al., 2013). As other social norms, cultural norms, represent general expectations about appropriate behaviors in societal space (Sherif, 1936; McDonald and Crandall, 2015). As such, they are expected to be powerful determinants of prejudice regulation (Verkuyten, 2011; Guimond et al., 2013, 2014; Anier et al., 2018, 2019). However, to date, the psychological determinants underlying the causal influence of cultural norms on prejudice regulation remain poorly identified.

Taking the case of France as a prime example, the present paper aims to fill this gap by highlighting specific processes of prejudice regulation under the dominant French cultural norm, the *Laïcité* (i.e., French secularism). More specifically, the Laïcité is a socio-political concept framed by law which establishes normative prescriptions related to cultural and religious diversity in French society. However, in recent decades, the Laïcité is declined into two antinomic norms: The *Historic Laïcité*, a longstanding egalitarian norm, shares the social space with it amended form, the *New Laïcité*, an assimilationist norm fostering social uniformity (Akan, 2009; Baubérot, 2012; Policar, 2017; Blanc, 2018). On top of this, the New Laïcité is related to higher levels of prejudice as compared to the Historic Laïcité (Kamiejski et al., 2012; Roebroeck and Guimond, 2015, 2017, 2018; Troian et al., 2018). To understand this relationship, research to date favors an “indirect-endorsement” path, examining how Laïcité endorsement produces distinct outcomes on intergroup attitudes. In parallel, recent experimental findings draw another possible path, showing that the mere contextual salience of these norms shapes prejudice and discrimination behavior beyond their endorsement (Anier et al., 2018, 2019). However, what is lacking is the identification of the socio-cognitive processes that can settle the causal explanation between both Laïcité and prejudice. We aim to fill this gap by arguing that the Laïcité norms can set the stage for specific prejudice regulations via a “direct-contextual” path.

## The French Cultural Context

### From the French Republican Model to the Laïcité Norms

In order to gauge how Laïcité influences prejudice regulation, one can start by situating the French ideological landscape with regard to past work on diversity ideologies. In the literature, diversity ideologies are generally classified according to two broad orientations (Plaut et al., 2018; Leslie et al., 2020): On the one hand, one finds (1) “identity-blind ideologies” like the assimilationist ideology requiring minorities to abandon their cultural identity for the benefit of a unique national identity. This category also includes the colorblind ideology, which prescribes the ignorance of group identity in favor of thinking of individuals as unique entities. On the other hand, one finds (2) “identity-conscious ideologies” such as multiculturalism ideology^3^ which values both the maintenance of cultural identity and the adoption of a common national culture (Wolsko et al., 2000; Berry, 2005, 2006; Guimond et al., 2010, 2014; Rosenthal and Levy, 2010; Levin et al., 2012). Within this category, research is recently also increasingly interested in polycultural ideology, which does not value the recognition of group differences *per se*, but instead the creation of mixed and malleable identities resulting from the contact between different cultures (Rosenthal and Levy, 2010; Morris et al., 2015; Pedersen et al., 2015; Grigoryev et al., 2018).

In fact, extensive comparative research suggests that when “identity-conscious ideologies” are favored as cultural norms in a given country, they shape more positive intergroup attitudes as compared to when “identity-blind ideologies” are favored as cultural norms. More specifically, Multiculturalism (e.g., the cultural norm in Canada) is mostly negatively associated with prejudice toward minorities. Conversely, assimilationism (e.g., the cultural norm in Germany) is positively linked to prejudice (Wolsko et al., 2000; Levin et al., 2012; Guimond et al., 2013; Whitley and Webster, 2019; Leslie et al., 2020). Concerning colorblindness (e.g., the cultural norm in the United States), although its initial professed goal is equality, the available findings suggest a complex pattern. Indeed, colorblindness is negatively related to prejudice when it is measured directly (e.g., self-report), but positively associated with it when measured indirectly (e.g., using measures which are less prone to social desirability; Wolsko et al., 2000; Richeson and Nussbaum, 2004; Norton et al., 2006; Apfelbaum et al., 2008; Vorauer et al., 2009; Plaut et al., 2018; Yogeeswaran et al., 2018).

With respect to these main ideological orientations, the French diversity ideology, coined *Republican Universalism*, on the whole, promotes an “identity-blind” approach as it values the transcendence of group affiliations for the benefit of a cohesive citizenship system. However, it appears to be endowed with a dual and antagonist ideological nucleus (Kamiejski et al., 2012; Guimond et al., 2014; Badea et al., 2015). Its first nucleus component is strongly assimilationist (Maisonneuve and Testé, 2007; Guimond et al., 2010; Sabatier et al., 2016). However, its second nucleus component, termed *Universalism* ensures citizen equality independently of any cultural or religious particularisms (French constitution, Art.1, 1958). Thus, scholars equate this latter universalism component to the original egalitarian goal of colorblind ideology (Badea, 2012; Guimond et al., 2014; Badea and Aebischer, 2017; Roebroeck and Guimond, 2018), and at times to a form of multiculturalism as it promotes tolerance toward minorities and cultural particularisms (Maisonneuve and Testé, 2007; Mahfud et al., 2016). These parallelisms reveal that the universalist component is more equality oriented than the assimilationist component. Therefore, it is expected to produce more favorable outcomes on intergroup relations than the assimilationist component (Badea et al., 2015). Yet, it should be noted that neither colorblindness nor multiculturalism are perceived as the prevalent cultural norms of diversity in France (Anier et al., 2019). In fact, this assimilation/universalism co-encapsulation gives rise to two specific cultural norms: the egalitarian Historic Laïcité and the assimilationist New Laïcité (i.e., following the terminology used in the social psychological literature; Roebroeck and Guimond, 2015; Nugier et al., 2016; Anier et al., 2019). Noteworthy, French citizens endorse more strongly the Historic and/or the New Laïcité than any other diversity ideology (Kamiejski et al., 2012; Anier et al., 2019). Consequently, this high degree of support for both Laïcité suggests that they are perceived as the prevalent ways to deal with diversity in French society or, in other words, as the cultural norms. Therefore, these two norms are particularly likely to be predictive of intergroup relations within the French context (Guimond et al., 2013).

### Historic and New Laïcité: Two Norms to Deal With Diversity

Since the French Revolution, the Laïcité^4^ represents a major institutionalized societal tool at the service of the French model to manage ethno-cultural diversity. However, because the Laïcité is framed by law, its normative frame is sensitive to the chain of socio-political events. For instance, during the 80s, French society underwent a social crisis marked by an increase in ethno-religious claims linked to the immigration waves from the later nineteenth century (Bistolfi, 2014; Gautherin, 2014; Machelon, 2015; Baudouin and Portier, 2018). To accompany these social mutations, political leaders revised the original juridical bases of the Laïcité. As a consequence, increasingly since 2004, the Laïcité is repeatedly marshaled in speeches concerning the place of Islam in French society. While at the same time, the defenders of the more traditional Laïcité do not hesitate to voice concerns about its ideological shift (Akan, 2009; Baubérot, 2012; Mangeot et al., 2012). Importantly, Kamiejski et al.’s (2012) seminal work on the French Laïcité showed that, at the psychological level, there are indeed, not one but at least two different conceptions of Laïcité that coexist in social space^5^ : the Historic and the New Laïcité.

In order to gauge the cultural specificity of both Laïcité norms and, in particular their differences, it seems necessary to engage in a short definitional analysis. In fact, the Laïcité prescriptions rest on three basic components (Constitutional Council, 2013): (1) *The state neutrality component* (i.e., the notion of state-religion separation): Within the Historic Laïcité, religious neutrality is limited to public officials (e.g., state agents, hospital agents, teachers etc.; Gautherin, 2014; Machelon, 2015; Policar, 2017). Nonetheless, a recent New Laïcité prescription prohibited visible religious symbols in middle schools (Education code Act no. 228, 2004), and since 2010, the display of any religious clothing that covers the head is forbidden in the public realm (Penal Code Act no. 1192, 2010). These laws suggest an extension of neutrality, from state institutions to the public space, and concomitantly, from state officials to everyday citizens (Bouillon, 2014; Gautherin, 2014; Policar, 2017; Saillant-Maraghni, 2017; Portier, 2018). (2) *The citizens’ fundamental freedoms component*: The Historic Laïcité values the freedoms of conscience, religion choice, and religious practice “individually or collectively, in the public and in the private area” (State Council, 1989). However, the New Laïcité aims to constrain the scope of these freedoms. As one can read in a document from the government’s Observatoire de la Laïcité (2016): “We must distinguish freedom of conscience and freedom of religious expression. […] The freedom of religious expression ought to be restrained to guarantee the respect of public order” (p. 3). And finally, (3) *the citizen equality component*: For the Historic Laïcité, equality is synonymous to non-discrimination (Redor-Fichot, 2005; Gautherin, 2014; Zuber, 2018). Specifically, it prohibits “access to educational settings based on the beliefs or religious beliefs of students” (State Council, 1989). This aspect is not so salient within the New Laïcité prescription. For example, the relatively recent exclusion of a high school girl who refused to take off her veil seems at odds with the inclusionary ideal of the Historic Laïcité (Gautherin, 2014). In sum, the Historic Laïcité norm is an equality norm used to fend off discrimination on the basis of cultural and religious particularities, while the New Laïcité is an assimilationist norm fostering social uniformity by neutralizing distinctive identity cues in the social space.

In fact, research confirms that these distinct normative orientations influence differently attitudes toward minorities. More specifically, a handful of studies show that the endorsement of Historic Laïcité is negatively linked to prejudice and when rendered salient decreases discrimination toward Maghrebians (i.e., the group which is most affected by racism in France; Pettigrew and Meertens, 1995; Dambrun and Guimond, 2001). Conversely, the endorsement of the New Laïcité norm is positively linked to prejudice toward these minorities and when rendered salient increases discriminatory behavior (Kamiejski et al., 2012; Roebroeck and Guimond, 2017; Anier et al., 2019). These results support the notion that Laïcité norms contribute to the cultural dynamics of intergroup relations in France. Thus, to understand these effects, the challenge is to smoothly articulate how these cultural factors interact with more general psychological determinants.

## From the Two Laïcité Norms to Prejudice: an Indirect-Endorsement Path

### Laïcité as a Legitimizing Myth

Seminal research on the Laïcité norms approached the issue from the perspective of interindividual variability in order to grasp: (1) the psychological underpinnings associated with the endorsement of either Historic or New Laïcité, and (2) the way through which this personal endorsement influences prejudice. To this purpose, Social Dominance Theory (SDT; Pratto et al., 1994; Sidanius and Pratto, 2001) was mobilized. This classic account assumes that individuals vary in their social dominance orientation (SDO), reflecting their degree of support for group-based hierarchies in society. Moreover, the SDO level shapes the endorsement of *Legitimizing Myths* namely beliefs or ideologies which enhance or attenuate existing hierarchical dynamics (Sidanius and Pratto, 2001). As such, high-SDO individuals gravitate toward Hierarchy Enhancing Legitimizing Myths (HE-LM), whereas low-SDO individuals favor Hierarchy Attenuating Legitimizing Myths (HA-LM). A fundamental property of SDT is that these endorsements predict, in turn, the level of prejudice (for a review, see Sibley and Duckitt, 2008). In other words, the relation between SDO and prejudice is mediated by the type of endorsed myth.

Crucially, diversity ideologies are considered as legitimizing myths (Levin et al., 2012; Guimond et al., 2014). For instance, high-SDO individuals are more likely to endorse an assimilationist ideology^6^ (serving a HE-LM function) and this endorsement, in turn, positively predicts prejudice. Whereas, low-SDO individuals are more likely to favor egalitarian ideologies, such as multiculturalism (serving a HA-LM function), and this endorsement negatively predicts prejudice (Levin et al., 2012; Guimond et al., 2013; Rattan and Ambady, 2013). Thus, in light of SDT, an emerging hypothesis is that the two Laïcité norms represent cultural legitimizing myths in France (Roebroeck and Guimond, 2017; Troian et al., 2018). Hence, depending on their SDO Level, French citizens will either slant toward the egalitarian Historic Laïcité to enable its HA-LM function, or toward the assimilationist New Laïcité to capitalize on its HE-LM function.

However, in the relevant literature, the available empirical data only partially support this contention. Concerning Historic Laïcité, the findings indicate, as expected, that low-SDO individuals endorse it more strongly. In turn, Historic Laïcité endorsement is negatively correlated to prejudice (Kamiejski et al., 2012; Roebroeck and Guimond, 2017). Nevertheless, none of these authors verified that Historic Laïcité endorsement mediates the SDO-prejudice relationship, which would constitute cogent evidence for ascribing it a legitimizing myth function (Sidanius and Pratto, 2001). Concerning the New Laïcité, SDO does not predict its endorsement (Kamiejski et al., 2012) or weakly so (below 0.20; Roebroeck and Guimond, 2017, 2018), while its endorsement is indeed positively correlated to prejudice. This unanticipated absence of a SDO-New Laïcité link led Troian et al. (2018) to suspect measurement issues in past studies. By developing their own New Laïcité measurement tool, they uncovered the predicted mediation: They found that higher SDO levels are associated with stronger New Laïcité endorsement which, in turn, predicts a higher level of prejudice. At the same time, these authors did not replicate past results concerning Historic Laïcité. Thus, a comprehensive test of these two norms operating as legitimizing myths is still needed.

### Laïcité as a Malleable Ideology?

Going one step further, personal attitudes toward Laïcité are also studied from the theory of malleable ideology (Knowles et al., 2009). This theory assumes that ideologies possess a certain degree of malleability, rather than a stable content as conceptualized by SDT. In fact, according to their SDO motives (i.e., hierarchy-enhancing vs. hierarchy-attenuating), individuals can alter the meaning of an ideology to match their personal goals. For instance, Knowles et al. (2009) showed that high-SDO individuals divert the original egalitarian purpose of colorblindness to legitimize intergroup inequality. Based on this rationale, Roebroeck and Guimond (2018) showed that in France the meaning of Laïcité is indeed diverted depending on individual’s SDO motives. First of all, they found that SDO is negatively related to Laïcité attachment (i.e., worded in an unqualified, generic manner), suggesting that it is originally an egalitarian ideology. However, in a situation of symbolic threat, high-SDO individuals reported an increase in general Laïcité attachment, while concurrently exhibiting a strong endorsement of the New Laïcité. Hence, these results suggest that, in specific contexts, high-SDO individuals construct the meaning of Laïcité no longer in its egalitarian conception but infusing it with the assimilationist elements of New Laïcité.

Taken together, research conducted from the perspective of SDT and malleable ideology frameworks provide interesting insights into the psychological determinants enabling individuals to adhere to the specific content of either the Historic or the New Laïcité. However, within this general “indirect-endorsement” path, the Laïcité norms were mainly measured and not manipulated. In fact, the way they can causally influence intergroup attitudes or responses in social settings is not directly addressed.

## From the Two Laïcité Norms to Prejudice: a Direct-Contextual Path to Probe

### A Direct-Contextual Influence?

To understand how the Laïcité norms shape intergroup attitudes in social settings, it is important to examine their influence beyond personal endorsement. Therefore, we surmise that just as any other prominent social norm, the Laïcité should be able to drive regulation processes via a direct-contextual path. Interestingly, Monteith and Walters (1998) showed that the way in which individuals construct the meaning of an ideology can also influence prejudice regulation. In particular, high-prejudiced individuals who conceived egalitarianism in terms of *equality of opportunity* (i.e., equality based on fair distribution of resources and opportunity) feel a moral obligation to regulate their prejudice and thus adopt low prejudice standards (e.g., not to appear prejudicial). Conversely, high-prejudiced individuals who conceive egalitarianism in terms of *individualism* (i.e., equality based on individual merits) do not exert such control on their prejudiced attitudes. These results are consistent with extensive research on modern racism showing that, at least from the 1980s, the global anti-prejudice discourse is associated with a strong social disapproval and legal punishment of racism in the public sphere (Gaertner and Dovidio, 1986; Devine, 1989; Blanchard et al., 1991, 1994; Pettigrew and Meertens, 1995; Sears and Henry, 2003). Accordingly, individuals developed motivations to control prejudice either to avoid the cost of these social sanctions (i.e., external goals) or to remain consistent with one’s own egalitarian values (i.e., internal goals; Devine, 1989; Plant and Devine, 1998; Brauer et al., 2000). Consequently, the salience of equality and anti-racism norms affect the ways individuals prevent prejudice in social settings beyond personal endorsement (Devine, 1989; Blanchard et al., 1994; Monteith et al., 1996; Wyer et al., 1998; Lowery et al., 2001; Crandall et al., 2002; Bodenhausen et al., 2009).

Concerning the Laïcité norms, a potential hint at this direct contextual influence may be spotted in the analysis of Roebroeck and Guimond (2017). They found that when SDO is statistically controlled, both Laïcité norms account, in and of themselves, for a distinct portion of variance in prejudice. Specifically, the Historic Laïcité is associated with a decrease in prejudice disclosure, while the New Laïcité is associated with an increase in prejudice disclosure. More recently, Anier et al. (2019) showed that the salience of Historic Laïcité decreases discrimination, while the salience of New Laïcité increases it. However, what is lacking in previous research is the identification of the socio-cognitive processes that can sustain a causal explanation between both Laïcité and prejudice. To fill this gap, we apply the Justification-Suppression Model (JSM; Crandall and Eshleman, 2003) to the French context.

### The Justification-Suppression Model in the French Context

The central idea of the JSM is that prejudice is not directly expressed, it goes instead through a regulatory filtering which either impedes or facilitates its expression. The starting point of the model is that within a global egalitarian climate, individuals are motivated to avoid prejudicial labels (Gaertner and Dovidio, 1986; Devine, 1989; Blanchard et al., 1991, 1994; Pettigrew and Meertens, 1995; Sears and Henry, 2003). This motivation is expected to drive self-regulation of prejudice via a well-known process of mental control: suppression (Wegner and Erber, 1992; Macrae et al., 1994; Wegner, 1994; Wenzlaff and Wegner, 2000). Suppression is put in motion to prevent undesirable thoughts from emerging into consciousness because they are judged to be inappropriate (Macrae et al., 1994; Wyer et al., 1998). One of the core assumptions of the JSM is that when individuals are immersed into a suppression context, they experience a motivational conflict. As stated by Crandall and Eshleman (2003), the JSM can be conceived as “a general model for how tension and equilibrium are reached within individuals between prejudice suppression and expression” (p. 433). This quote outlines that conflict arises from two antagonistic motivations: One that pushes for the expression of prejudice that comes to mind and another that urges to suppress prejudice because its expression is prohibited.

The originality of the JSM resides in its proposition that under distinct circumstances this conflict can be resolved by the covert expression of prejudice driven by justification processes. Justification is defined as an intentional strategy to seek contexts or situations allowing an innocuous and unsanctioned way of expressing prejudice. Justification is triggered by an individual’s motivation to release the tension induced by the act of suppression, while preserving the self-image as unprejudiced. Hence, justifications can be any belief, value, or ideology that can serve as a convenient explanation to release prejudice in social settings. This last consideration indicates a second fundamental property of the JSM. Indeed, Crandall and Eshleman (2003) propose that most individual attitudes or beliefs (e.g., political orientation, values, or religious systems) defined in the literature as antecedents of prejudice can be conceived to operate as potential suppression or justification factors. Based on this, a social norm such as Laïcité appears as a plausible instigator of both suppression and justification processes.

**FIGURE 1:**
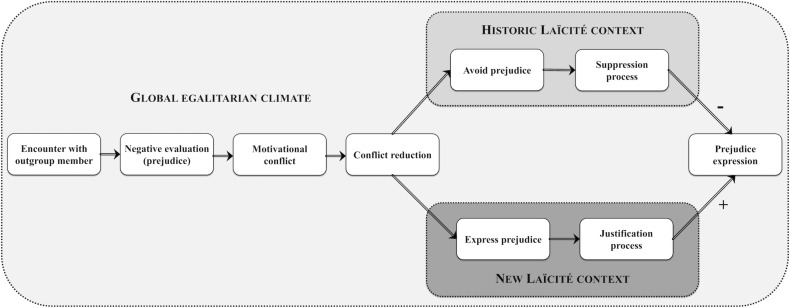
Schematic representation of the hypothesized model of prejudice expression within the French cultural-ecological context as a function of salient Laïcité norms. The encounter of an outgroup member activates negative prejudiced thoughts which trigger a motivational conflict due to their incongruity with the global egalitarian climate. This conflict can be resolved via two regulation routes: (a) when Historic Laïcité is salient, avoid prejudice via the suppression process, or (b) when New Laïcité is salient, express prejudice via the justification process.

By applying this general reasoning to the specific French ecological-cultural context, it is expected that when Historic Laïcité is salient individuals should be motivated to protect their social and/or self-image from being labeled as racist. It is expected that these social motives drive self-regulation of prejudice via suppression. Conversely, under an assimilationist context embodied in the New Laïcité, individuals should be motivated to release the pressure induced by the continuous demands to suit egalitarianism. It is expected that this covert and rationalized expression of prejudice is driven by justification (see Figure 1). On this basis thereof, we now turn to examine the empirical evidence that supports these innovative hypotheses by highlighting their operational functioning. Furthermore, through the JSM prism we discuss the effectiveness of the two Laïcité in their potential to ensure harmonious intergroup relationships.

## Historic Laïcité and Intergroup Relations: the Suppression Pathway

### Evidence for Historic Laïcité as a Context of Suppression

To investigate whether an egalitarian norm such as the Historic Laïcité is a genuine context of suppression, one can turn to the specific operating principles of this process. According to classic models of mental control (Wegner and Erber, 1992; Bargh, 1994; Macrae et al., 1994; Wegner, 1994), when an individual engages in thought suppression two processes are put in motion: (a) a controlled operating process that replaces the unwanted thoughts with distractors, and (b) an automatic monitoring process that scans the content of the cognitive system in search of unwanted thoughts. Both processes work in synergy: The detection of unwanted thoughts by the monitoring process recruits the operating process. Thus, a successful cycle of suppression casts out unwanted thoughts from consciousness therefore reducing their public manifestation (Wegner and Erber, 1992; Wegner, 1994). However, this cycle is known to generate ironic consequences on subsequent cognition and behavior (for a review, see Monteith et al., 1998a). In fact, it is assumed that during the cycle of suppression the repeated detection (and thus activation) of stereotypic thoughts leads to their hyperaccessibility (Macrae et al., 1994; Galinsky and Moskowitz, 2007). As a consequence, when the demand of suppression is relaxed, the activated unwanted thoughts tend to color subsequent judgments to a greater extent than if suppression had never occurred (Macrae et al., 1994; Wyer et al., 1998). This initial reduction followed by a subsequent increase in stereotyping has been coined a *rebound effect*.

In fact, research indicates that the control of prejudice when egalitarian and anti-racist norms are salient is driven by suppression (Macrae et al., 1994; Monteith et al., 1998a; Wyer et al., 1998; Shelton, 2003; Bodenhausen et al., 2009). Furthermore, scholars argue that this spontaneous suppression is also driven by specific cultural norms such as a colorblind norm in the United States (Wolsko et al., 2000; Richeson and Nussbaum, 2004; Norton et al., 2006; Apfelbaum et al., 2008; Todd and Galinsky, 2012). Indeed, the normative prescription of colorblind emphasizing racial myopia invites individuals to suppress group labeling. More specifically, research highlights that in a colorblind context, individuals control the overt expression of prejudice (e.g., its self-reported form). However, this negative link between colorblind and prejudice could be only apparent (Wolsko et al., 2000; Richeson and Nussbaum, 2004; Norton et al., 2006; Apfelbaum et al., 2008; Correll et al., 2008; Todd and Galinsky, 2012; Plaut et al., 2018). For instance, Correll et al. (2008) highlighted that following a colorblind prompt, participants initially express low level of prejudice, but following a time delay, they show an increase in prejudice report (i.e., a rebound effect) suggesting that individuals regulate prejudice expression via suppression (see also Wolsko et al., 2000).

By analogy, in France the Historic Laïcité is the major egalitarian norm to fend off racism (Redor-Fichot, 2005; Gautherin, 2014; Zuber, 2018). As such, its contextual salience in social space could lead individuals to commit themselves to an “identity-blind” mindset and thus suppress prejudice. To date, the findings in the literature reveal that Historic Laïcité is negatively correlated to overt prejudice disclosure (Kamiejski et al., 2012; Roebroeck and Guimond, 2017), and when rendered salient it causes a decrease in discrimination (Anier et al., 2019). These results are consistent with those found when prejudice is assessed directly (e.g., with explicit measures of prejudice) in the realm of egalitarian norms or a colorblind ideology^7^. Building on this finding, we argue that a convenient way of testing the Historic Laïcité suppression hypothesis would be to experimentally introduce a subsequent measure of prejudice expression (following an initial measure) to uncover a rebound effect. This type of index, without being exhaustive, opens up new research perspectives to highlight the operation of suppression in the realm of Historic Laïcité. Furthermore, if the existing data seem to indicate that the Historic Laïcité can be a promising route for prejudice reduction, our analysis suggests that the picture might be more complex than originally assumed.

### Intergroup Relations in the Context of Historic Laïcité

The suppression of prejudice prevents its social expression but does not actually reduce prejudice itself as illustrated by the rebound effect. What is more, the unexpected consequences of suppression are not limited to this classic phenomenon. For example, research indicates that during suppression, majority members experience aversive and tense states partly due to the motivational conflict described by Crandall and Eshleman (2003) (see also Devine et al., 1991; Monteith et al., 1993; Monteith, 1996). Furthermore, they show signs of behavioral avoidance during intergroup interactions (e.g., less eye contact; Norton et al., 2006; Trawalter and Richeson, 2006). Yet, other research suggests that these consequences are nor inevitable nor automatic.

In fact, upon a closer look, studies indicate that the suppression of prejudice toward socially sensitive groups (e.g., African Americans in the United States) does not systematically lead to a rebound effect (Monteith et al., 1998b; Gordijn et al., 2004). Indeed, the mere presence of a target of a normatively protected group can, in and of itself, operate as a reminder and reactivate equality standards (Lowery et al., 2001; Castelli and Tomelleri, 2008). And, as long as egalitarian norms are salient, individuals are expected to pursue the goal to avoid prejudice. Thus, this suggests that under ecological situations (e.g., a global egalitarian climate), even after an initial suppression period, this activated goal should prevent a rebound effect (Sedikides, 1990; Thompson et al., 1994; Ford and Kruglanski, 1995; Dumont and Yzerbyt, 2001; Gordijn et al., 2004). However, it also suggests that individuals will look for ways to bypass the tension induced by the continuous demands to suit egalitarianism (Crandall and Eshleman, 2003). Therefore, this analysis leads us to mitigated conclusions concerning the effectiveness of the Historic Laïcité to favor harmonious relations. Specifically, it is still possible that within this context individuals are motivated to release the pressure of suppression via the justification process.

## New Laïcité and Intergroup Relations: the Justification Pathway

### Evidence for the New Laïcité as a Context of Justification

This assimilationist New Laïcité appears as a cultural norm likely to be an acceptable context to release prejudice via the justification process. In fact, the notion that within a global egalitarian normative climate, individuals rely on beliefs, norms or ideologies to legitimate/justify prejudice is found in many theoretical accounts (for a review, see Costa-Lopes et al., 2013) such as classic treatments on prejudice (Allport, 1954; Gaertner and Dovidio, 1986), as well as within the System justification theory (SJT; Jost and Banaji, 1994), or even SDT (Sidanius and Pratto, 2001). However, a valuable contribution of the JSM is that it describes certain operational indicators to assign a justification function to a given factor. More specifically, the first sine qua non indicator is to uncover a positive correlation between a suspected justification factor and prejudice. Furthermore, the second fundamental indicator is to show that the manipulation of the suspected justification factor produces an increase in prejudice beyond any personal endorsement. Based on the JSM, the available empirical evidence on the effects of the New Laïcité on intergroup attitudes concurs with these two indicators.

Indeed, a strong correlational link between New Laïcité and prejudice is found in at least six studies using 10 independent samples (Kamiejski et al., 2012; Roebroeck and Guimond, 2015, 2017; Nugier et al., 2016; Troian et al., 2018; Anier et al., 2018, 2019). Moreover, Nugier et al. (2016, study 2) already suggested that the New Laïcité is used to justify prejudice according to the JSM framework. For instance, they showed that high-prejudiced individuals rated more negatively a Muslim target exhibiting a deviant behavior according to the New Laïcité prescriptions (e.g., a woman claiming her right to wear the veil) as compared to a deviant catholic target (e.g., a woman claiming her right to wear a cross). Interestingly, these results suggest that it is not the deviant behavior *per se* that is sanctioned, rather the group membership of the target. Finally, Anier et al. (2019) showed that the manipulation of the New Laïcité norm causes an increase in discriminatory behavior. Taken together, these results fit the hypothesis that the prescriptions of the New Laïcité could constitute a broad context to justify prejudice toward minorities.

However, this assumption requires additional convergent empirical demonstrations. For instance, Crandall and Eshleman (2003) explain that the justification process is responsible for a reduction in the gap between direct (i.e., self-reported) and indirect (i.e., covert) indicators of prejudice. In other words, the level of prejudice expressed directly should be aligned with the one expressed indirectly when a justification is at stake. Based on this, an interesting research perspective could reside in the joint measurement of prejudice (using direct and indirect measures) following the manipulation of a New Laïcité ideological prompt vs. a control condition.

### Intergroup Relations in the Context of New Laïcité

The present analysis questions at its root the beneficial contribution of a cultural norm such as the New Laïcité to the social ideals of acceptance and harmonious intergroup coexistence. Indeed, the New Laïcité appears as an institutional and social framework which allows an unsanctioned justification of prejudice while preserving a favorable self-image. In fact, Crandall and Eshleman (2003) argue that when justification is enabled, the motivation for expression is thus satisfied without any threat of a social sanction. In line with this, they argue that the justification of prejudiced views may have positive hedonic consequences. As a result, through positive reinforcement, this could encourage individuals to reiterate the expression of prejudice via this process.

Furthermore, we consider that the promotion of New Laïcité principles can be used as broad arguments to justify prejudice. For example, the New Laïcité was recently wielded by politicians as a privileged rhetorical tool against gender-related discrimination (Stasi, 2003; Redor-Fichot, 2005). At the same time, one can read on a press release of the Laïcité Observatory (2016): “The reservations are mainly expressed with regard to women’s clothing. Hostility or reservation are linked to the feeling of symbolic aggression by the religious expression perceived as proselytizing in the collective space” (p. 4). Hence, and somewhat ironically, under the guise of the fight against sexism, the Muslim religion is specifically targeted. Moreover, past studies showed that the New Laïcité was not only linked to prejudice against Muslims but also to the North-African community altogether (Kamiejski et al., 2012; Roebroeck and Guimond, 2017). That being said, the fact of systematically assimilating the Muslim religion with people of North African origin is itself a cultural stereotype. From this standpoint, it is more generally argued that the fight against sexism, the visible symbols of the Muslim religion, or the condemnation of deviant acts (Nugier et al., 2016) are only a handful of the manifold sub-arguments derived from the New Laïcité to justify prejudice against Maghrebian culture.

## Scope of the Model

Overall, the analysis of the French context shows that political and social mutations generate shifts in cultural norm meanings as it is presently the case for Laïcité. In fact, these shifts are not specific to the French cultural-context as illustrated by the malleability of color blindness in the United States (Knowles et al., 2009). Moreover, as argued by Guimond (2011), in Germany and in the United Kingdom, political actors make use of the term “multicultural ideology” to discuss about cultural segregation phenomena (i.e., the non-adoption of the host culture by some minority groups). As such, the original meaning of multiculturalism ideology is diverted (see Berry, 2005, 2006). These examples nicely illustrate how political rhetoric and intergroup context can participate in the fluctuating meanings of cultural norms. In fact, our working model offers an integrative analysis grid to account for the consequences of these cultural normative shifts on intergroup attitudes. Indeed, via an “indirect-endorsement path,” the coexistence of distinct cultural norm meanings can be reinforced and used to fit individuals’ motives (Sidanius and Pratto, 2001; Knowles et al., 2009; Guimond et al., 2013), and via a “direct-contextual path” they can influence the ways individuals regulate prejudice (Gaertner and Dovidio, 1986; Devine, 1989; Crandall and Eshleman, 2003). Importantly, the analysis of this “direct-contextual path” in the French context shows that it is necessary to be particularly vigilant to the framing of egalitarian and anti-racism norms within societies. For example, the egalitarian Historic Laïcité is used in the socio-political discourse as a bulwark against the normative drifts of the New Laïcité (Akan, 2009; Mangeot et al., 2012; Gautherin, 2014). However, our detailed analysis of the socio-cognitive processes suggests that the Historic Laïcité frame could drive prejudice suppression and that it is not de facto an effective way to reduce prejudice itself. As a consequence, although egalitarian norms are propagated in society with the noble intentions to fight against racism, their framing may be sometimes inefficient if the ultimate objective is to foster harmonious intergroup relations.

Yet, research demonstrates that the ideological frame of “identity-conscious ideologies” such as multiculturalism can be an efficient route to reduce prejudice itself (Wolsko et al., 2000; Levin et al., 2012; Guimond et al., 2013; Whitley and Webster, 2019; Leslie et al., 2020). In fact, when the similarities and differences with outgroups are highlighted, this can drive the regulation of prejudice via yet another process: perspective-taking (i.e., an active attempt to embrace and identify with the experience of another individual; Todd et al., 2011). Indeed, perspective-taking reduces prejudice, increases recognition of inequalities and produces more positive intergroup interactions (Galinsky and Moskowitz, 2000; Todd et al., 2011; Todd and Galinsky, 2012).

Applied to the French context, this analysis suggests that the norm of Historic Laïcité could achieve its goal of promoting social equality if it is properly framed as an “identity-conscious” cultural norm in the political and social discourse. This idea does not appear merely as an abstract consideration because research suggests that French citizens actually embrace some aspects of multicultural ideology. For instance, both majority and minority members sometimes express a preference for integration (i.e., a strategy intrinsically related to multiculturalist ideology; Berry, 2006) rather than assimilation and sometimes an equal preference between the two acculturation strategies (Maisonneuve and Testé, 2007; Kamiejski et al., 2012). Furthermore, from the minority standpoint, this endorsement of the integration strategy is related to positive attitudes toward the Historic Laïcité. What is more, from the majority standpoint, the endorsement of Historic Laïcité is positively related to the endorsement of multiculturalist ideology (Kamiejski et al., 2012). Taken together, these findings are encouraging as they indicate that the Historic Laïcité is somewhat associated with an increased tolerance toward the preservation of minority cultures. Going beyond the French cultural-ecological context, the present analysis suggests that the effectiveness of equality norms in combating racism depends on how their prescriptions are framed, disseminated, and negotiated within society as a whole.

## Conclusion

In French society, two distinct Laïcité norms are used as sociopolitical tools to handle diversity. To understand their effects on intergroup attitudes, we complemented the existing “indirect-endorsement” explanation (Kamiejski et al., 2012; Roebroeck and Guimond, 2017; Troian et al., 2018), with an analysis of their “direct-contextual” influence. Specifically, we argue that the desire to appear non-prejudiced drives the suppression of prejudice within the realm of the egalitarian Historic Laïcité norm. Conversely, the desire to release the pressure stemming from a relentless commitment to egalitarianism encourages the justification of prejudice within the realm of the assimilationist New Laïcité context. Of course, additional cogent evidence is needed to empirically substantiate these hypotheses. Furthermore, we discuss the implications of these processes on the effectiveness of both Laïcité to favor harmonious relationships. We suggested that beyond the specific case of Laïcité, such a causal model could be used as an interpretation framework for understanding intergroup dynamics in other cultural-ecological contexts. Specifically, future research could be dedicated to examine the conditions under which specific cultural norms may trigger suppression vs. justification, or even other regulation processes (e.g., perspective-taking).

In a nutshell, we embrace the idea that integrative attempts are required to understand the complex nature of intergroup attitudes (Duckitt, 1992; Cuddy et al., 2009; Guimond et al., 2013). In fact, research would gain in predictive power by taking into account the context-sensitivity to explain variations in prejudice within and across countries, and the ways it shapes the expression of cultural stereotypes (Verkuyten, 2011; Guimond et al., 2013, 2014; Anier et al., 2018, 2019; Roebroeck and Guimond, 2018). Indeed, prejudice, cultural stereotypes and discriminatory behaviors know no geographic nor temporal boundaries. However, their targets, their content and their forms fluctuate at the pendulum of sociopolitical mutations across places. In this process, we hope that the present contribution will constitute an insightful analysis to reveal possible stereotyping dynamics within and across countries in the constellation of their ideological correlates.

## Author Contributions

L-AL and TA drafted the manuscript together and approved the final version of the manuscript for submission. Both authors contributed to the theoretical development of the ideas presented in the manuscript.

## Conflict of Interest

The authors declare that the research was conducted in the absence of any commercial or financial relationships that could be construed as a potential conflict of interest.

## Footnotes

1 According to the classic tripartite model of attitudes prejudice and stereotypes are interrelated as they represent two facets of the same construct. Specifically, prejudice is the affective component and stereotype is the cognitive component of group attitude (Rosenberg and Hovland, 1960; Eagly and Chaiken, 1993; Fiske, 1998). From a functional perspective, this relationship is viewed as an interactive process. Indeed, when cultural stereotypes serve to explain ingroup/outgroup differences (e.g., via a justification function), they are driven by a pre-existing prejudice (Allport, 1954; Crandall and Eshleman, 2003; Park and Judd, 2005; Crandall et al., 2011). In other words, prejudice constitutes a prerequisite as it is subsequently rationalized via the expression of cultural stereotypes. Hence, gauging the downstream consequences of prejudice regulation on the expression of cultural stereotypes is essential to understand their persistence in modern societies.

2 Guimond et al. (2013) use the term *cultural norm of integration*. However, as pointed out by Anier et al. (2018), this term can generate confusion with respect to research on acculturation (Berry, 2005, 2006). In this article we therefore opt for the term “cultural norm of diversity.”

3 Multiculturalism is a term used to label different phenomena in the literature (Yogeeswaran and Dasgupta, 2014; Grigoryev et al., 2020; Verkuyten and Yogeeswaran, 2020). For example, it may refer to: (a) a demographic situation (i.e., a general depiction of a population characterized by cultural diversity); (b) a diversity ideology; and (c) a cultural norm. Taking this into account, it is important to note that most of the experimental work which is cited in this paper is carried out in the United States context. As such, it explores multiculturalism as a diversity ideology because it is not the official cultural norm (Guimond et al., 2013; Yogeeswaran et al., 2018). Indeed, when multiculturalism is studied as a cultural norm, this implies to take also into account a more detailed analysis of the acculturation process. More specifically, according to Berry’s Model (2005, 2006) the acculturation process describes how the majority and minority groups become acculturated through mutual contact. In a given society, when minorities seek to maintain some aspect of their original culture while at the same time adopting the culture of the host country by participating in social life, they opt for an “integration strategy.” At the societal level, when this strategy is also favored by the dominant group and for instance, encouraged by legal policies (e.g., as in Canada), it is referred as a “multiculturalist cultural norm.” In sum, to count as a cultural norm, multiculturalism must act at the societal level, and provide a generic reference for intergroup relations.

4 To the extent that Laïcité is a sociopolitical concept with a legal framing, it differs from the concept of secularism which is generally defined as the progressive disappearance of religious thinking within society (Hayat, 2006; Baubérot, 2012).

5 It should be noted that one exception in the literature concerns the work of Cohu et al. (2018), which studies beliefs in Laïcité as a polysemic and multidimensional construct rather than a stable dual structure.

6 Note that studies indicate that the link between SDO and assimilation is not straightforward. It may depend on how individuals define the assimilationist ideology (Thomsen et al., 2008; Guimond et al., 2010).

7 The connection between Historical Laïcité and Colorblindness is only conceptual. These two cultural norms share a common “identity-blind” root to achieve an equality ideal. However, in the context of the Historic Laïcité, the state is oblivious to religion (e.g., for instance, there is no official religion in France) to allow every citizen to be free to follow its cultural and religious standards. As such, the individual and the social space do not have to be neutral *per se* (Redor-Fichot, 2005; Gautherin, 2014; Zuber, 2018). While the colorblind ideology prescribes social dismissal of ethnic identities at the institutional and at the individual levels (Yogeeswaran et al., 2018).
